# Automatic thoughts mediate the relationship between social appearance anxiety and attitudes toward cosmetic surgery

**DOI:** 10.1186/s40359-026-04740-x

**Published:** 2026-05-21

**Authors:** Erkan Durar, Turgut Şöhret, Cafer Bakaç

**Affiliations:** 1 Department of Nursing, Faculty of Health Sciences, Iğdır University, Iğdır, 76103 Turkey; 2 Faculty of Nursing, Department of Surgical Diseases Nursing, Atatürk University, Erzurum, 25050 Turkey; 3 TUM School of Management, Technical University of Munich, Munich, 80333 Germany

**Keywords:** Automatic thoughts, Cosmetic surgery, Nursing students, Social appearance anxiety

## Abstract

Previous studies have examined the relationship between social appearance anxiety and attitudes toward cosmetic surgery. However, the psychological mechanisms underlying this relationship remain relatively underexplored. Drawing on cognitive perspectives suggesting that appearance-related anxiety may activate negative self-referent cognitions, the present study examined automatic thoughts as a potential mediating mechanism linking social appearance anxiety to attitudes toward cosmetic surgery. Data were collected from 251 nursing students (167 females; Mage = 21.24, SD = 2.22). Mediation analyses indicated that automatic thoughts significantly mediated the relationship between social appearance anxiety and attitudes toward cosmetic surgery. These findings contribute to the literature by highlighting a cognitive pathway that may help explain how social appearance anxiety is associated with attitudes toward cosmetic surgery. Empirical and practical implications of the findings are discussed.

**Supplementary Information:**

The online version contains supplementary material available at 10.1186/s40359-026-04740-x.

## Introduction

The increasing pressure on physical appearance in society is causing individuals to develop concerns about their social image. With the widespread use of social media and digital platforms, individuals are increasingly exposed to idealized body images. As a result, there has been a noticeable rise in anxiety related to physical appearance [1]. Nursing students who will work in the healthcare sector may be particularly affected by this issue. Their future professional roles involve direct interaction with patients and the public. In addition, they are continuously exposed to societal appearance norms and ideal body images through social media [2].

Social appearance anxiety (SAA), known as a type of social anxiety, is defined as anxiety that arises when individuals evaluate their body and physical appearance negatively and fear negative evaluation from others based on their appearance [3, 4]. Individuals experiencing such concerns may resort to various strategies to alleviate their appearance-related anxiety. One of these strategies may include cosmetic surgery. Individuals who worry about their social appearance due to poor body image are more likely to seek cosmetic surgical procedures to change their physical appearance [5].

Attitudes toward cosmetic surgery refer to individuals’ evaluative beliefs and predispositions regarding the acceptance or consideration of surgical procedures intended to modify physical appearance. Several studies have reported that the most significant factors prompting individuals to consider cosmetic surgery are social appearance anxiety, body dissatisfaction, and automatic thoughts [5–8].

Although there is accumulating evidence relating social appearance anxiety to attitudes toward cosmetic surgery, literature examining the psychological mechanisms underlying this relationship remains limited. Social appearance anxiety refers specifically to the fear of negative evaluation by others based on one’s overall physical appearance in social contexts. It is conceptually distinct from body dissatisfaction, which reflects a person’s negative evaluation of their own body or specific body features [9, 10]. Recent theoretical models describe social appearance anxiety as an interpersonal-evaluative construct. In contrast, body dissatisfaction is considered a self-evaluative body-image outcome [11, 12].

It is also important to note that social appearance anxiety and automatic thoughts are conceptually and empirically distinct constructs. Social appearance anxiety is a trait-like and affectively laden construct reflecting a chronic fear of negative evaluation by others based on physical appearance. Therefore, it is primarily interpersonal and evaluative in nature [3, 4]. Automatic thoughts, in contrast, are not stable traits but cognitive process variables. They are rapid, involuntary, and situation-triggered cognitions that represent negative self-referential interpretations activated in response to stressors [13].

Combining evidence from several research traditions, automatic thoughts may represent an important mechanism linking social appearance anxiety to attitudes toward cosmetic surgery. Automatic thoughts may shape individuals’ emotional responses to social anxiety, body dissatisfaction, and aesthetic concerns. They may also play an intermediary role in the formation of attitudes and behaviors [14]. In the present model, social appearance anxiety represents the social-evaluative trigger. Body dissatisfaction represents evaluative content. Automatic thoughts represent the proximal cognitive mechanism linking these experiences to surgery-related attitudes.

Based on these considerations, the present study aims to extend previous literature by investigating the mediating role of automatic thoughts in the relationship between social appearance anxiety and attitudes toward cosmetic surgery. By doing so, the study contributes to the literature by examining automatic thoughts as a psychological mechanism that may explain how social appearance anxiety translates into attitudes toward cosmetic surgery. The study was conducted with a group of nursing students. Focusing on nursing students also provides an important empirical contribution, as this population may be particularly sensitive to appearance-related concerns due to both social pressures and professional role expectations. In this way, the research contributes to both the literature and practice in several ways. Empirically, the study provides evidence that automatic thoughts may function as a psychological mechanism linking social appearance anxiety to attitudes toward cosmetic surgery. Practically, the findings highlight nursing students as a potentially vulnerable group in terms of social appearance anxiety due to both individual body perception and professional performance concerns [15].

Supporting this perspective, a study conducted with university students reported that 82.08% of students experienced body image anxiety. More than 75.65% of male students and 88.00% of female students reported being affected by this issue. Additionally, a negative correlation was found between the frequency of body image anxiety and mental health levels [16].

### Hypotheses development

Cosmetic surgery is a specialized field concerned with the preservation, reconstruction, or improvement of an individual’s physical appearance, thereby contributing to an enhanced quality of life [5]. The prevalence of cosmetic surgery in the general population has increased, particularly due to the rising interest of young individuals in altering their physical appearance through cosmetic surgery [5]. According to data from the International Society of Aesthetic Plastic Surgery (ISAPS) in 2024, the number of surgical procedures performed for aesthetic purposes worldwide was reported to be over 17 million, and the number of non-surgical procedures to be over 20 million. As a result, nurses are now encountering more patients seeking cosmetic surgical procedures in hospitals [17]. Thus, it is of critical importance to know about how patients approach aesthetic surgery, how this approach tends to change over time, and the factors associated with this trend as this would be beneficial for nursing students, who are future nurses, in planning quality nursing care for this special patient group [2].

Nurses are dedicated to enhancing the health of individuals, families, and communities, preventing diseases, and restoring health through care [18]. Consequently, it is imperative for nurses to maintain a comprehensive state of psychosocial and spiritual well-being. Elevated psychosocial well-being among nurses is crucial for the advancement and improvement of both individual and community health. Additionally, nursing students encounter unique challenges, including academic demands, clinical training, and professional expectations. Contemporary models of professional identity and social evaluative anxiety suggest that individuals in training-based, performance-observed professions are particularly susceptible to appearance-related self-evaluations and cognitive distortions. These factors can exacerbate social appearance anxiety, especially given the significance of maintaining a professional appearance in healthcare settings [11]. Previous research has demonstrated that dissatisfaction with one’s body and concerns about appearance can adversely affect mental health, resulting in increased stress and diminished mental well-being [12]. Within cognitive-behavioral frameworks, the combination of high evaluation exposure and the development of professional identity may amplify automatic negative self-thoughts, rendering nursing students a theoretically informative population for examining cognitive mediation mechanisms. Furthermore, comprehending the dynamics of social appearance anxiety is vital in nursing education, as it directly influences students’ ability to confidently engage in clinical settings, which is essential for effective patient care and professional development.

Not only these anxieties but also the consequences of these anxieties are fruitful areas for research endeavors as there is limited literature on this. Specifically, from the previous literature we know that social appearance anxiety and the attitudes toward cosmetic surgery (as a way to tackle the negative self-body-image) are positively associated [1, 5]. However, these studies are largely correlational and descriptive in nature and do not sufficiently explain the underlying psychological processes that translate appearance-related anxiety into favorable attitudes toward cosmetic surgery. Without a cognitive explanatory framework, the relationship remains theoretically under-specified and limited in its interpretive power. However, the evidence on the mechanisms and especially psychological mechanisms of this relationship is lacking. Therefore, a mediator model grounded in cognitive theory is needed to clarify how anxiety-related perceptions are transformed into evaluative and behavioral attitudes toward surgical appearance modification. Based on the previous literature [9, 10], automatic thoughts could play a mediating role in this relationship as individuals’ body-related social anxiety may result in negative self-image cognitions and emotions, which in turn, may be a reason why individuals view cosmetic surgery positively.

On a related matter, it should also be noted that according to classical cognitive theory [19], automatic thoughts are positioned as antecedents of emotional reactions, including anxiety. However, contemporary cognitive-affective frameworks [20, 21] recognize emotional states as activators and intensifiers of automatic cognitive processing, on which the present model draws on. Specifically, social appearance anxiety may serve as a distal affective context within which automatic thoughts are generated and sustained. Following this, social appearance anxiety may prime individuals toward negative self-referential appraisals when appearance-relevant cues are encountered. This ordering is also empirically supported by research showing that social evaluative anxiety precedes and predicts the content of automatic cognitions in interpersonal contexts [9, 22]. Therefore, in this model, we treat social appearance anxiety as a distal predictor and automatic thoughts as the proximal cognitive mechanism mediating its effect on attitudes toward cosmetic surgery. Based on these, we hypothesize:

- Hypothesis 1: Social appearance anxiety and attitudes toward cosmetic surgery are positively related.
- Hypothesis 2: Social appearance anxiety is positively associated with automatic thoughts.
- Hypothesis 3: Automatic thoughts are positively associated with attitudes toward cosmetic surgery.
- Hypothesis 4: Automatic thoughts mediate the relationship between social appearance anxiety and attitudes toward cosmetic surgery.

### Methods

#### Participants

Data were collected from a convenience sample of 251 nursing students (167 females; *M*_*age*_ = 21.24, *SD*_*age*_ = 2.22) enrolled at a large university in Türkiye in 2025. The rationale for this sample size was that correlations tend to stabilize when the sample size approaches 250 participants [23]. Of these participants, 14 (5.6%) indicated that they have previously received a psychiatric diagnosis; 66% indicated that their mothers were either illiterate or had primary education; 30.7% reported their father’s education level to be either illiterate or primary education. Finally, majority of the participants (81.7%) self-reported that their family had an average family income.

To evaluate whether our sample size was sufficient to detect the indirect effect observed in our mediation model (see in the Results section), we conducted a sensitivity power analysis for the indirect effect using the observed correlations and standard deviations among our study variables as proposed by Schoemann et al. [24]. The analysis indicated high power (1–β = 0.96).

### Measures

#### Social Appearance Anxiety (SAA) 

We used a Turkish adapted version of social appearance anxiety scale developed by Hart et al. and adapted to Turkish by Doğan to measure participants’ cognitive, emotional, and behavioral anxieties related to their own appearance. On 5-point Likert scale, participants were asked to indicate the extent to which each of the 16 items is characteristic for them 1 (“not at all”) to 5 (“extremely”). A sample item included “I get nervous talking to people because of the way I look” [25, 26]. In this study, the Cronbach’s alpha (α) was found to be .96.

#### Automatic Thoughts (AT)

A validated Turkish version (Şahin & Şahin,) of the Automatic Thoughts Questionnaire developed by Hollon and Kendall was utilized to measure the severity of negative thoughts and negative self-assessments. Although the ATQ was originally developed to assess automatic thoughts associated with depressive symptomatology [27], its use here is justified on both conceptual and empirical grounds. Conceptually, the ATQ captures the content and frequency of negative self-referential that are not exclusive to depression but co-occur with a broad range of negative emotional states, including social anxiety [9, 14]. At the same time, the validated Turkish version [28] has previously been used in different Turkish samples involving social anxiety and appearance-related constructs, which further supports its applicability here [9, 14]. For ATQ, the participants were instructed that they would read a variety (i.e., 30 items) of thoughts that pop into people’s heads and for each thought, indicate how frequently, if at all, the thought occurred to them over the last week from 1 (“not at all”) to 5 (“all the time”). The measure has five subscales, which are “Negative Self-Concept” (sample item: “I am worthless”), “Confusion and Escape Fantasies” (sample item: “I wish I were somewhere else”), “Personal Maladjustment and Desire for Change” (sample item: “My life is not going the way I want it to”), “Loneliness/Isolation” (sample item: “No one understands me”), and “Giving up/Hopelessness” (sample item: “I do not think I can go on”) [27, 28]. In this study, the Cronbach’s alpha (α) for the total scale was .98 and for the subscales were .94, .90, .78, .84, and .85 respectively.

#### Acceptance of Cosmetic Surgery (ACS) 

To measure participants’ attitudes toward cosmetic surgery, we used a validated Turkish version (Karaca et al.,) of acceptance of cosmetic surgery scale developed by Henderson-King and Henderson-King. For each of the 15 items, participants were asked to indicate the degree to which they agree or disagree with the item on 7-point Likert scale ranging from 1 (“strongly disagree”) to 7 (“strongly agree”). Higher scores on both the sub-dimensions and the total scale indicate more favorable attitudes toward cosmetic surgery. Three subscales are Personal (sample item: “Cosmetic surgery is a good thing because it can help people feel better about themselves”), Social (sample item: “I would seriously consider having cosmetic surgery if my partner thought it was a good idea”) and Consider (sample item: “I have sometimes thought about having cosmetic surgery”), which covers (1) motivations for cosmetic surgery and individuals’ personal evaluations regarding their appearance; (2) attitudes endorsing cosmetic surgery relating to individuals’ desire to feel better in social relationships and settings; and (3) individuals’ opinions regarding cosmetic surgery [29, 30]. In the present study, Cronbach’s alpha (α) for the total scale was .93, and were .90, .89, and .86 for the subscales, respectively.

#### Control variables 

We measured gender, age, psychiatric diagnosis, and income as covariates because these variables have been linked in prior research to social appearance anxiety, automatic thoughts, and attitudes toward cosmetic surgery. To illustrate, there are gender differences in terms of social appearance anxiety [31]. Additionally, Social appearance anxiety is associated with broader psychological symptoms that relate to negative/automatic cognitions [32]. Likewise, attitudes toward cosmetic surgery vary as a function of sociodemographic factors [33].

### Data collection procedure

Data were collected through a face-to-face survey. The surveys were administered directly to the participants by the researcher or research team, with questions presented in a standardized sequence. Responses were recorded on a survey form and subsequently digitized. Participation was entirely voluntary, and no incentives were provided to the participants. The principles of confidentiality and anonymity were strictly upheld.

### Analyses

The first step in the analysis was to confirm that the scales measuring the focal variables assessed distinct constructs. We conducted three confirmatory factor analyses (CFAs) with progressively differentiated factor structures: (1) all items from the SAA, AT, and ACS scales loading onto a single factor; (2) SAA and AT items loading onto one factor and ACS items onto a separate factor; and (3) each scale loading onto its own factor. Models were estimated using the lavaan package [34] in R and compared using the χ², CFI, TLI, RMSEA, and SRMR fit indices. We then assessed discriminant validity of these scales following the criteria outlined by Rönkkö and Cho [35], which holds when latent correlations between constructs are sufficiently low to conclude they represent distinct entities. Specifically, discriminant validity was evaluated using two complementary approaches. We examined whether the upper bound of the 95% confidence interval for each inter-factor latent correlation fell below |0.80|, which indicates no evidence of a discriminant validity problem, or below |.90|, which suggests the constructs are likely distinct [35].

To test the hypotheses, we conducted a mediation analysis with the social appearance anxiety as a predictor, automatic thoughts as a mediator, acceptance of cosmetic surgery as an outcome variable and gender, age, psychiatric diagnosis status and self-reported family income level as control variables. This analysis was planned a priori and conducted using total scores of the variables. However, we also exploratorily conducted several supplementary mediation analyses to examine if the proposed mediation analysis also holds for the subscales of the outcome variable as well as mediator, using the same approach. All of the analyses were conducted in R using the PROCESS macro for R [36]. Indirect effects were estimated with 5,000 bootstrap samples with 95% bias-corrected confidence intervals (CIs).

Raw data and analyses code could be found on Open Science Framework at this anonymous link: https://osf.io/aqzjt/overview?view_only=f25e6fe46e304271b63feb7adbc8079b.

## Results

### CFA and discriminant validity

CFA results (see Table 1) showed that the three-factor hierarchical model for items from SAA, AT, and ACS loading onto their own subfactors and subsequently, factor had a better model fit (χ2 = 3648, *df* = 1584, *CFI* = .98, *TLI* = .98, *RMSEA* = .05, *SRMR* = .07). The results for discriminant validity showed that the upper limit of the confidence interval of the correlation between social appearance anxiety and automatic thoughts was .63; of the correlation between social appearance anxiety and acceptance of cosmetic surgery was .41; and of the correlation between automatic thoughts and acceptance of cosmetic surgery was .48. These findings indicate no evidence for the problem of discriminant validity.

**Table 1:** Fit indices with different factor solutions for study variables

Model	χ^2^	df	Δdf	Δχ^2^	CFI	TLI	RMSEA	SRMR	AIC	BIC
One factor	85,867	1769	-	-	0.52	0.51	0.12	0.14	44,628	45,058
Two factors	6876	1768	-1	-1691***	0.65	0.64	0.11	0.12	42,919	43,353
Three factors	4629	1766	-2	-2247***	0.81	0.81	0.08	0.08	40,676	41,117
Three factors with subfactors	3648	1584	-182	-981***	0.98	0.98	0.05	0.07	38,438	38,886

Robust fit indices were reported

*df*degrees of freedom, *CFI*comparative fit index, *TLI*Tucker–Lewis index, *RMSEA*root mean square error of approximation, *SRMR*standardized root mean square residual, *AIC*Akaike information criterion, *BIC*Bayesian information criterion

*N* = 251. Factors = SAA, AT, and ACS, χ^2^ = chi-square

*** *p* < .001

### Descriptive statistics

The results indicated that social appearance anxiety is positively related to automatic thoughts (*r* = .54, *p* < .01), and acceptance of cosmetic surgery (*r* = .27, *p* < .01). Furthermore, automatic thoughts were positively associated with acceptance of cosmetic surgery (*r* = .33, *p* < .01). See Table 2 for further details and see Table 1 in Supplementary Materials for detailed relationships including subscales of automatic thoughts and acceptance of cosmetic surgery.

**Table 2:** Means, standard deviations, and correlations

Variable	M	SD	1	2	3	4	5	6
1. Gender (females = 0)	0.33	0.47						
2. Age	21.24	2.22	0.06					
3. Psychiatric diagnosis (yes = 1)	0.06	0.23	0.09	0.00				
4. Self-reported family income	2.01	0.43	− 0.19**	0.04	− 0.00			
5. Social appearance anxiety	36.70	13.25	0.01	− 0.04	0.08	− 0.01		
6. Automatic thoughts	67.87	27.07	0.04	− 0.06	0.09	− 0.09	0.54**	
7. Acceptance of cosmetic surgery	49.62	21.60	− 0.02	0.02	0.16*	− 0.05	0.27**	0.33**

M and SD are used to represent mean and standard deviation, respectively

*N* = 251

* indicates *p* < .05

** indicates *p* < .01

### Hypothesis testing

**Fig. 1:**
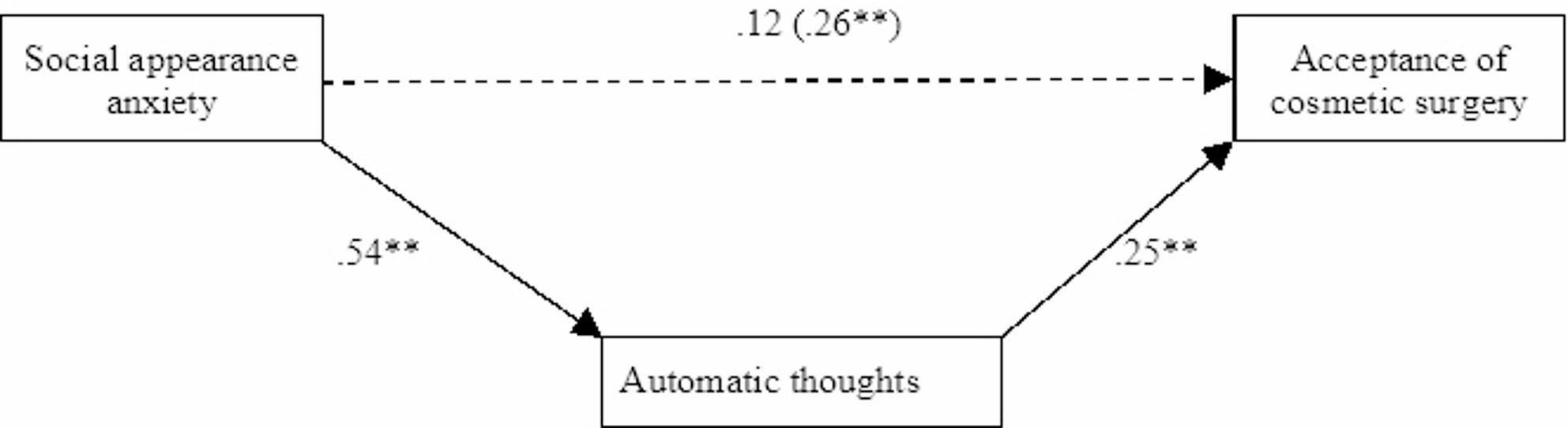
Mediation model for the influence of social appearance anxiety on acceptance of cosmetic surgery automatic thoughts. Note. *N* = 251. The value in parentheses represents the direct effect of social appearance anxiety on acceptance of cosmetic surgery without controlling for automatic thoughts. The values represent standardized regression coefficients. ** *p* < .01

Before hypothesis testing analysis, we checked whether the linearity assumption among variables held. The results supported the assumption of linearity for the relationships among variables. To test the hypotheses that social appearance anxiety and attitudes toward cosmetic surgery are positively related (Hypothesis 1); that social appearance anxiety is positively associated with automatic thoughts (Hypothesis 2); that automatic thoughts are positively associated with attitudes toward cosmetic surgery (Hypothesis 3); and automatic thoughts mediate the relationship between social appearance anxiety and attitudes toward cosmetic surgery (Hypothesis 4), we conducted a mediation analysis. The results showed a significant and positive relationship between social appearance anxiety and acceptance of cosmetic surgery (*b* = 0.42, *β* = 0.26, *t* = 4.23, *p* < .001; all *variance inflation factors (VIFs)* = 1.00), confirming Hypothesis 1. This effect did not remain significant after controlling for the effect of automatic thoughts (*b* = 0.20, *β* = 0.12, *t* = 1.73, *p* > .05). Furthermore, in line with Hypothesis 2 and 3, the results demonstrated a positive relationship between social appearance anxiety and automatic thoughts (*b* = 1.10, *β* = 0.54, *t* = 10.06, *p* < .001; all *VIFs* = 1.00), and positive association between automatic thoughts and acceptance of cosmetic surgery (*b* = 0.20, *β* = 0.25, *t* = 3.53, *p* < .001; all *VIFs* ranging from 1.00 to 1.40). Most importantly, the indirect effect of social appearance anxiety on acceptance of cosmetic surgery via automatic thoughts was significant (*indirect effect* = 0.22, *SE* = 0.08, *95% CI* [0.07, .39]). These results remained significant and similar after excluding the control variables. The details of the mediation model and a visual illustration of the indirect effect are provided on Table 3; Fig. 1, respectively.

**Table 3:** Regression results using automatic thoughts (mediator) and acceptance of cosmetic surgery (outcome variable) as the criterion

Predictor	Automatic Thoughts				Acceptance of Cosmetic Surgery			
	b(SE)	t	β		b(SE)	t	β	
Intercept	46.90(15.93)**	2.94			24.24(14.44)	1.68		
Gender (females = 0)	1.08(3.12)	0.34	0.02		-2.56(2.78)	-0.92	− 0.06	
Age	-0.49(0.65)	-0.76	− 0.04		0.41(0.58)	0.71	0.04	
Psychiatric diagnosis (yes = 1)	6.23(6.30)	0.99	0.05		11.93(5.63)*	2.12	0.13	
Self-reported family income	-4.73(3.42)	-1.38	− 0.07		-2.07(3.06)	-0.67	− 0.04	
Social appearance anxiety	1.10(0.11)**	10.06	0.54		0.20(0.11)	1.73	0.12	
Automatic thoughts	-	-	-		0.20(0.06)**	3.54	0.25	
*F*								
*R* ^*2*^	0.31				0.14			

*b* represents unstandardized regression weights

*β* indicates the standardized regression weights

*N* = 251

*SE* Standard Error

* indicates *p* < .05

** indicates *p* < .01

In addition to these, we also tested the indirect effects of social appearance anxiety on acceptance of cosmetic surgery via automatic thoughts for the three subfacets of acceptance of cosmetic surgery (Personal, Social and Consider). For these subfacets, the indirect effects were similar to that of the total scale and were significant (Personal: *indirect effect* = 0.07, *SE* = 0.03, *95% CI* [0.01, 0.14]; Social: *indirect effect* = 0.07, *SE* = 0.03, *95% CI* [0.02, 0.14]; Consider: *indirect effect* = 0.08, *SE* = 0.03, *95% CI* [.02, .14]).

## Discussion

This study started with the idea that social appearance anxiety is positively related to the extent to which individuals have positive attitudes toward cosmetic surgery, and that automatic thoughts play a mediating role in this relationship. Overall, we found supporting evidence for our hypotheses that (1) there is a positive relationship between social appearance anxiety and attitudes toward cosmetic surgery, (2) social appearance anxiety and automatic thoughts are positively associated, (3) automatic thoughts and attitudes toward cosmetic surgery are positively associated and (4) finally and most importantly, automatic thoughts mediate the effect of social appearance anxiety on attitudes toward cosmetic surgery. Beyond appearance-focused frameworks, these findings are also consistent with contemporary cognitive models emphasizing that situation-triggered automatic cognitions function as proximal regulators of emotional responses and downstream evaluative attitudes [20, 37]. Recent cognitive–affective regulation models indicate that automatic self-referential appraisals play a central role in translating social-evaluative threat into coping-oriented attitudes and intentions [20]. In this sense, the present findings extend previous research by empirically demonstrating that automatic thoughts may operate as a cognitive mechanism through which appearance-related social concerns translate into favorable attitudes toward cosmetic surgery. While prior studies have documented direct associations between these variables, our findings suggest that the relationship may also involve an intermediary cognitive process that shapes how individuals interpret and respond to appearance-related social threats. Taken together, these findings highlight the potential role of cognitive mechanisms in explaining how appearance-related social concerns translate into evaluative attitudes toward cosmetic surgery.

Following the findings previous literature [5, 13], we hypothesized a positive relationship between social appearance anxiety and attitudes toward cosmetic surgery. The findings showed support for this hypothesis. This finding is in line with the previous literature showing similar relationship between the variable of interest. Specifically, this finding confirms the findings from previous studies showing a positive relationship between individuals’ social appearance anxiety and their attitudes toward cosmetic surgery, with social appearance anxiety related to poor body image being the main reason for preferring cosmetic surgery [1, 3, 5, 13]. Contemporary self-related emotion regulation literature further suggests that when appearance-based self-threat is activated, individuals may engage in compensatory self-enhancement or self-modification strategies to regulate negative affect and self-discrepancy signals [20, 21]. The results might also indicate that an individual’s interactions with their social environment, media influence, and societal beauty norms may shape their attitudes toward cosmetic surgery. Specifically, experimental research has demonstrated that exposure to retouched social media imagery can influence thin-ideal internalization and the acceptance of cosmetic surgery among young women, particularly when image manipulation is explicitly disclosed [38]. Taken together, these findings suggest that social appearance anxiety may function as a contextual trigger that activates broader self-evaluative concerns, which in turn increase the attractiveness of appearance-modifying solutions such as cosmetic surgery. Beyond these theoretical explanations, it is also important to consider how such dynamics may operate within specific populations. In this sense, nursing students might be especially prone because at work, they may be exposed to a large presence of social appearance anxiety from patients, which may in turn increase their dissatisfaction with their own bodies. This, further, may lead them to view interventions such as cosmetic surgery more favorably. However, this assumption should be tested empirically.

Additionally, we hypothesized that social appearance anxiety and automatic thoughts are positively related. We found evidence for this hypothesis, too. This finding is also consistent with the findings from previous literature [9, 14, 22]. Specifically, the finding highlights that social appearance anxiety may be related to how others may evaluate these individuals, which may result in individuals’ negative thoughts, such as loneliness, hopelessness, personal incompatibility, confusion, and avoidance, about themselves. Current cognitive-affective processing and self-referential thinking models conceptualize such automatic thoughts as fast, schema-driven appraisals that amplify negative affect and bias interpretation under social threat conditions [20, 21, 37]. In other words, the findings support the idea that social appearance anxiety may activate maladaptive cognitive schemas related to self-worth and social acceptance, thereby increasing the frequency of automatic negative thoughts. From a broader cognitive–affective perspective, these results also align with models suggesting that social-evaluative threats may activate schema-driven cognitive responses that shape individuals’ emotional reactions. Similarly, it is possible that individuals’ negative thoughts about themselves may result in anxiety that others may evaluate based on their appearance. This, however, is yet to be tested with longitudinal data.

Additionally, the results demonstrated confirming evidence for a positive relationship between automatic thoughts and attitudes toward cosmetic surgery. This finding reveals that individuals’ internal patterns of thought can influence their body image and perspectives on aesthetic interventions. The positive relationship observed in the study indicates that nursing students who experience more intense automatic thoughts develop more favorable attitudes toward cosmetic surgery. This suggests that individuals may seek to alter their appearance in an effort to cope with negative body image, low self-esteem, or feelings of inadequacy. Within contemporary self-regulation frameworks, this pattern can be interpreted as cognition-driven coping, in which maladaptive automatic thoughts increase reliance on external, appearance-focused regulation strategies rather than internal cognitive restructuring [20, 21]. Indeed, literature frequently addresses the relationship between body image disturbance and automatic thoughts; it emphasizes that these thoughts can create dissatisfaction with physical appearance and increase the tendency toward cosmetic interventions [39, 40]. Thus, the present findings suggest that automatic thoughts may serve not only as a reflection of negative self-evaluations but also as a cognitive pathway that shapes individuals’ openness to appearance-altering solutions. In this sense, automatic thoughts may represent a proximal cognitive process through which broader emotional and self-evaluative concerns translate into attitudes toward cosmetic interventions. Following this, the finding is consistent with the idea that psychological variables such as body image, stress, or the pursuit of social acceptance may influence attitudes toward cosmetic surgery.

Finally, and most importantly, we hypothesized a mediating role of automatic thoughts in the relationship between social appearance anxiety and attitudes toward cosmetic surgery and found evidence supporting this hypothesis. This finding provides preliminary support for the idea that automatic thoughts may act as a cognitive bridge connecting appearance-related social anxiety to evaluative attitudes toward cosmetic surgery. To our knowledge, and based on the literature we reviewed, few studies have explicitly examined automatic thoughts as a potential cognitive mechanism linking social appearance anxiety to attitudes toward cosmetic surgery; therefore, this contribution should be considered preliminary rather than definitive. This mediation pattern is broadly consistent with contemporary cognitive mediation and emotion regulation models, which propose that distal social-evaluative stressors may influence attitudes and behavioral preferences through proximal self-referential cognitive processes [19, 21]. In this respect, the present study contributes to the literature by highlighting the potential role of cognitive processes—specifically automatic thoughts—in shaping how individuals translate appearance-related social pressures into attitudes toward cosmetic interventions. More broadly, these findings underscore the importance of considering cognitive pathways when examining how social and emotional experiences influence appearance-related attitudes. This is an important limitation because the anxiety that individuals may experience as a result of thinking that others may evaluate their appearance may bring about automatic thoughts for these individuals, which may lead them to be more open regarding cosmetic surgery. To be sure, existing studies indicate a significant relationship between social appearance anxiety and attitudes toward cosmetic surgery [5, 13] and show that automatic thoughts serve as a cognitive bridge between anxiety and attitude in this process [9], which are also supported by this study. Furthermore, we were not only interested in the mediation effect but also wanted to go beyond that and examine if the mediation effect holds for different subfacets of attitudes toward cosmetic surgery, which are Personal, Social and Consider. We found a similar mediation effect for all these subfacets, with these effects being smaller than the one for the total scale. Taken together, these results suggest that the proposed pathway reflects a broader cognitive–emotional regulation mechanism extending beyond appearance-specific domains [19, 21, 37, 41].

### Theoretical and practical implications

Despite modest effect sizes, this study has several theoretical and practical implications. From a theoretical perspective, this study extends the previous literature by positioning automatic thoughts as a mediating mechanism for the relationship between social appearance anxiety and attitudes toward cosmetic surgery. By doing so, we posit that when individuals’ anxieties related to how they may be evaluated by others might result in automatic thoughts about themselves. These negative thoughts, in turn, may trigger individuals to search for possibilities to alter their appearance in line with the social demands and thus, favorable views and attitudes toward cosmetic surgery. Additionally, automatic thoughts can be regarded just one psychological mediator among many others that are yet to be examined. From a practical perspective, these findings are particularly important as they demonstrate how individuals studying in health fields can have their cognitive processes influence their aesthetic perceptions and attitudes. Thus, nursing education should not be limited only to imparting professional knowledge and skills, but should also encompass students’ self-awareness, body image, and psychosocial functioning. The modifiability of automatic thoughts within cognitive behavioral therapy underscores the significance of structured psychoeducational and CBT-based programs in interventions aimed at nursing students’ mental health. These programs may encompass modules on identifying appearance-related automatic thoughts, cognitive restructuring exercises, stress and social-evaluative anxiety management skills, and guided self-monitoring through thought records. Additionally, brief group-based workshops integrated into nursing education—incorporating psychoeducation, cognitive restructuring practice, and reflective discussions on body image and professional identity—may assist students in critically evaluating appearance-based beliefs and attitudes toward cosmetic surgery. In the context of nursing education, these findings suggest the potential value of cognitive awareness and metacognitive skills training to enhance the understanding of nursing students’ aesthetic attitudes and to facilitate early, targeted intervention when maladaptive thought patterns are detected.

### Limitations and future studies

This study has several limitations. First, the data used to test the hypotheses were derived from a sample of nursing students. Given their background knowledge of the health risks associated with cosmetic surgery, their attitudes toward cosmetic surgery may differ from those of individuals in other professions. Consequently, to enhance the generalizability of the results, future research should collect data from a broader range of professional backgrounds. Second, and most critically, the study employed a cross-sectional design, which precludes causal inferences. As all variables were measured at a single time point using self-report instruments, the findings reflect only associative patterns and should not be construed as evidence of causal or directional effects. To establish more robust causal and directional claims regarding the relationships between the variables of interest, future studies could induce social appearance-related anxieties and observe their impact on automatic thoughts and attitudes toward cosmetic surgery. Experimental and longitudinal designs are essential for testing temporal ordering and causal pathways among variables. Additionally, the direction of the relationship between social appearance anxiety and automatic thoughts could also be reversed, suggesting that individuals’ negative self-perceptions may lead to anxieties about others’ evaluations of their appearance. Given the cross-sectional self-report design, reciprocal and bidirectional effects cannot be excluded from the present study. To address this, longitudinal data could be collected from participants on the variables in question to examine the circular effects that these variables exert on each other. This would additionally circumvent the risk of common method bias, which may be present in our data as all of the variables in our model were measured at one point in time. Third, other variables such as internalization of aesthetic ideals, the use of social media, self-esteem, and body mass index (BMI) are variables that have conceptual links to attitudes toward cosmetic surgery. Owing to this, it is not surprising that at least part of the indirect effect could be attributed to these unmeasured confounders. Thus, future studies could integrate these variables into their mediation models. Fourth, although the hierarchical confirmatory factor analysis employed in this study was theoretically justified and yielded acceptable model fit, this approach introduces structural complexity that may not generalize across different populations or cultural contexts. To tackle this, future research should seek to validate this measurement structure in more diverse samples.

## Conclusion

This study contributes to the literature by providing preliminary evidence that automatic thoughts may function as a psychological mechanism through which social appearance anxiety relates to attitudes toward cosmetic surgery. As a result, the findings of the study indicate that nursing students’ automatic thoughts may represent an important variable associated with their attitudes toward cosmetic surgery. In this regard, it is recommended to include psychosocial education content involving body image, self-esteem, and cognitive awareness in nursing education programs. In particular, developing skills to cope with automatic thoughts may be important for students to achieve both individual well-being and to carry out their professional roles in a healthier manner.

## Supplementary Information

Supplementary Material 1.

## Data Availability

The datasets analyzed for the current study are available on this anonymous link: [https://osf.io/aqzjt/overview?view\_only=f25e6fe46e304271b63feb7adbc8079b](https:/osf.io/aqzjt/overview?view_only=f25e6fe46e304271b63feb7adbc8079b)

## Code availability

Codes for data analyses for the current study are available on this anonymous link: https://osf.io/aqzjt/overview?view_only=f25e6fe46e304271b63feb7adbc8079b.
