# Supplementary material for: Automatic thoughts mediate the relationship between social appearance anxiety and attitudes toward cosmetic surgery
Source: BMC Psychol. 2026 May 21;14:1111. doi: 10.1186/s40359-026-04740-x (PMC13412126; doi:10.1186/s40359-026-04740-x)
Supplement: Supplementary file 1 — Supplementary Material 1. [file 40359_2026_4740_MOESM1_ESM.doc]

Supplementary Materials for
Automatic Thoughts Mediate the Relationship between Social Appearance Anxiety and Attitudes toward Cosmetic Surgery

Table of Contents
Table 1	1


Table 1 Means, standard deviations, and correlations 
 Variable	M	SD	1	2	3	4	5	6	7	8	9	10	11	12	
1. Gender (females = 0)	0.33	0.47	 	 	 	 	 	 	 	 	 	 	 	 	
2. Age	21.24	2.22	.06	 	 	 	 	 	 	 	 	 	 	 	
3. Psychiatric diagnosis (yes = 1)	0.06	0.23	.09	.00	 	 	 	 	 	 	 	 	 	 	
4. Self-report family income	2.01	0.43	-.19**	.04	-.00	 	 	 	 	 	 	 	 	 	
5. Social appearance anxiety	36.70	13.25	.01	-.04	.08	-.01	 	 	 	 	 	 	 	 	
Automatic Thoughts															
6. Negative self-concept	20.16	9.07	.06	-.02	.08	-.08	.59**	 	 	 	 	 	 	 	
7. Confusion and escape fantasies	14.46	6.24	.02	-.08	.11	-.05	.47**	.84**	 	 	 	 	 	 	
8. Personal maladjustment and desire for change	7.74	3.08	.00	-.06	.00	-.10	.34**	.72**	.83**	 	 	 	 	 	
9. Loneliness/Isolation	9.54	3.84	.01	-.10	.09	-.10	.52**	.82**	.84**	.77**	 	 	 	 	
10. Giving up/Helplessness	9.13	4.08	.02	-.06	.08	-.08	.48**	.84**	.87**	.82**	.84**	 	 	 	
Acceptance of Plastic Surgery															
11. Personal	19.06	8.65	-.07	-.01	.07	.00	.09	.16*	.19**	.20**	.19**	.20**	 	 	
12. Social	13.03	7.72	.12	.04	.18**	-.14*	.40**	.41**	.36**	.24**	.33**	.36**	.58**	 	
13. Consider	17.53	8.53	-.09	.02	.17**	-.01	.22**	.26**	.24**	.20**	.30**	.26**	.67**	.63**	
Note. N = 251. M and SD are used to represent mean and standard deviation, respectively. 
* indicates p < .05. ** indicates p < .01.
